# Identification of a glycolysis-related lncRNA prognostic signature for clear cell renal cell carcinoma

**DOI:** 10.1042/BSR20211451

**Published:** 2021-08-27

**Authors:** Wei Ma, Manli Zhong, Xiaowu Liu

**Affiliations:** 1 Department of Breast Surgery, The First Affiliated Hospital of China Medical University, Shenyang, China; 2 College of Life and Health Sciences, Northeastern University, Shenyang, China; 3 Department of Urology, The First Affiliated Hospital of China Medical University, Shenyang, China

**Keywords:** clear cell renal cell carcinoma (ccRCC), glycolysis, long noncoding RNAs (lncRNAs), prognosis, risk model

## Abstract

**Background:** The present study investigated the independent prognostic value of glycolysis-related long noncoding (lnc)RNAs in clear cell renal cell carcinoma (ccRCC).

**Methods:** A coexpression analysis of glycolysis-related mRNAs–long noncoding RNAs (lncRNAs) in ccRCC from The Cancer Genome Atlas (TCGA) was carried out. Clinical samples were randomly divided into training and validation sets. Univariate Cox regression and least absolute shrinkage and selection operator (LASSO) regression analyses were performed to establish a glycolysis risk model with prognostic value for ccRCC, which was validated in the training and validation sets and in the whole cohort by Kaplan–Meier, univariate and multivariate Cox regression, and receiver operating characteristic (ROC) curve analyses. Principal component analysis (PCA) and functional annotation by gene set enrichment analysis (GSEA) were performed to evaluate the risk model.

**Results:** We identified 297 glycolysis-associated lncRNAs in ccRCC; of these, 7 were found to have prognostic value in ccRCC patients by Kaplan–Meier, univariate and multivariate Cox regression, and ROC curve analyses. The results of the GSEA suggested a close association between the 7-lncRNA signature and glycolysis-related biological processes and pathways.

**Conclusion:** The seven identified glycolysis-related lncRNAs constitute an lncRNA signature with prognostic value for ccRCC and provide potential therapeutic targets for the treatment of ccRCC patients.

## Introduction

Renal cell carcinoma (RCC) is a common lethal urologic malignancy with over 430000 new cases and approximately 180000 deaths per year, accounting for roughly 2% of cancer diagnoses and cancer-related deaths worldwide [1,2]. Clear cell (cc) RCC (ccRCC) is the predominant subtype of RCC (75% of cases) [3] and has poor prognosis, especially for patients with advanced and metastatic disease, with a 5-year survival rate that is less than 12% [4]. As such, there is an urgent need for novel drug target and prognostic biomarkers for ccRCC that can improve disease diagnosis and patient survival.

Changes in energy metabolism are a biochemical hallmark of cancer. Anaerobic glycolysis has long been regarded as the primary metabolic process for energy production and anabolic growth in cancer cells [5], which is known as the Warburg effect [6]. The reprogramming of metabolic activities by cancer cells leads to cancer progression [7]. There is increasing evidence that metabolic changes—especially glycolysis—are closely related to the occurrence and development of ccRCC [8,9], offering a potential avenue for therapeutic targeting.

Long noncoding (lnc) RNAs (lncRNAs) are a class of nonprotein-coding RNA transcripts longer than 200 nucleotides [10,11] that are associated with multiple biological and pathologic processes; for instance, lncRNAs have been implicated in cancer initiation, progression, and metastasis [11,12] and facilitate cancer progression and predict poor prognosis [13,14]. However, it is not known whether these effects are related to the modulation of glycolysis by lncRNAs in ccRCC.

In the present study, we analyzed lncRNA expression data in ccRCC from The Cancer Genome Atlas (TCGA) and identified prognostic lncRNAs associated with glycolysis in ccRCC cells. We then established and validated an lncRNA signature for ccRCC based on seven of the identified glycolysis-related lncRNAs for predicting the survival of ccRCC patients.

## Materials and methods

### Patient data acquisition

Clinical and pathologic information and RNA sequencing data of 539 ccRCC patients were obtained from TCGA (https://cancergenome.nih.gov/). Gene expression data were standardized using the edgeR package of R software [15]. To increase the accuracy of the analysis, 507 cases with complete follow-up information and survival time >30 days were screened for subsequent analyses. The clinical characteristics of the study population are shown in Table 1.

**Table 1:** Clinical pathological parameters of ccRCC patients

Parameter	Number (*n*=507)	Percentage (%)
Age (years)		
≤60	260	51.3
>60	247	48.7
Gender		
Female	174	34.3
Male	333	65.7
Grade		
G1	12	2.4
G2	215	42.4
G3	199	39.2
G4	73	14.4
Gx	5	1.0
Unknown	3	0.6
Stage		
I	253	49.9
II	53	10.4
III	116	22.9
IV	82	16.2
Unknown	3	0.6
T		
T1	259	51.1
T2	65	12.8
T3	172	33.9
T4	11	2.2
N		
N0	225	44.4
N1	16	3.1
Nx	266	52.5
M		
M0	401	79.1
M1	78	15.4
Mx	26	5.1
Unknown	2	0.4

Abbreviations: M, distant metastasis; N, lymph node; T, tumor.

### Identification of glycolysis-related lncRNAs in ccRCC

A total of 457 glycolysis-related genes were extracted from the Molecular Signatures Database for gene set enrichment analysis (GSEA: M5937). Pearson correlation analysis was carried out to determine the correlation between lncRNAs and glycolysis-related genes based on glycolysis-related mRNA–lncRNA coexpression data using the limma package of R [16]. Finally, 297 glycolysis-related lncRNAs were identified based on a correlation coefficient > 0.6 and *P*<0.001.

### Establishment of a glycolysis-related lncRNA prognostic signature for ccRCC

Samples were randomly divided into training and validation sets at a 1:1 ratio. We screened glycolysis-related lncRNAs significantly associated with overall survival (OS) in the training cohort by univariate Cox proportional hazard regression analysis with *P*<0.001 as the cutoff. Glycolysis-related lncRNAs that were most highly correlated with OS were identified by least absolute shrinkage and selection operator (LASSO) regression using the glmnet package of R [17]. Significant lncRNAs for constructing the prognostic signature were identified by multivariate Cox regression analysis. We then used the following formula to calculate the risk score based on the expression levels of lncRNAs in each sample: risk score = exp1 × δ1 + exp2 × δ2…+ expn × δn, where expn is the expression level of each lncRNA, and δn is the regression coefficient in the multivariate Cox analysis for the target lncRNA. Using the median risk score in the training set, ccRCC patients from TCGA were divided into high- and low-risk groups. The difference in survival between the two groups was assessed by Kaplan–Meier survival analysis in the training, validation, and total cohorts using the survival and survminer packages of R [18,19].

### Independent prognostic analysis and receiver operating characteristic curve analysis

Univariate and multivariate Cox proportional hazard regression analyses were carried out to evaluate the correlations between survival outcome and clinicopathologic parameters and risk score in the total cohort using the survival package of R. To assess the predictive accuracy for survival time, we generated time-dependent receiver operating characteristic (ROC) curves for the training, validation, and total cohorts using the survival ROC package of R [20]. We then analyzed the relationship between the expression of glycolysis-related lncRNAs and clinicopathologic factors.

### GSEA

We identified different functional phenotypes between the high- and low-risk groups with GSEA v4.1.0 (https://www.broadinstitute.org/gsea/index.jsp) [21], and assessed the mRNA expression profiles of ccRCC patients from the TCGA dataset using Kyoto Encyclopedia of Genes and Genomes gene sets. Enriched gene sets with a nominal *P*<0.05 and false discovery rate < 0.25 after 1000 random sample permutations were considered statistically significant.

### Statistical analysis

Statistical analyses were performed with R v4.0.3 software [22]. The ggpubr package of R [23] was used to analyze the correlation between the expression levels of seven glycolysis-related lncRNAs and clinicopathologic factors. The high-dimensional data of the whole genome, 457 glycolysis-related genes, and risk model of seven glycolysis-related lncRNA expression profiles were subjected to principal component analysis (PCA) for dimension reduction, pattern recognition, and exploratory visualization [24,25]. GSEA was used for functional annotation. *P*<0.05 was considered statistically significant in all tests.

## Results

### Identification of glycolysis-related lncRNAs with prognostic value in ccRCC

**Figure 1:**
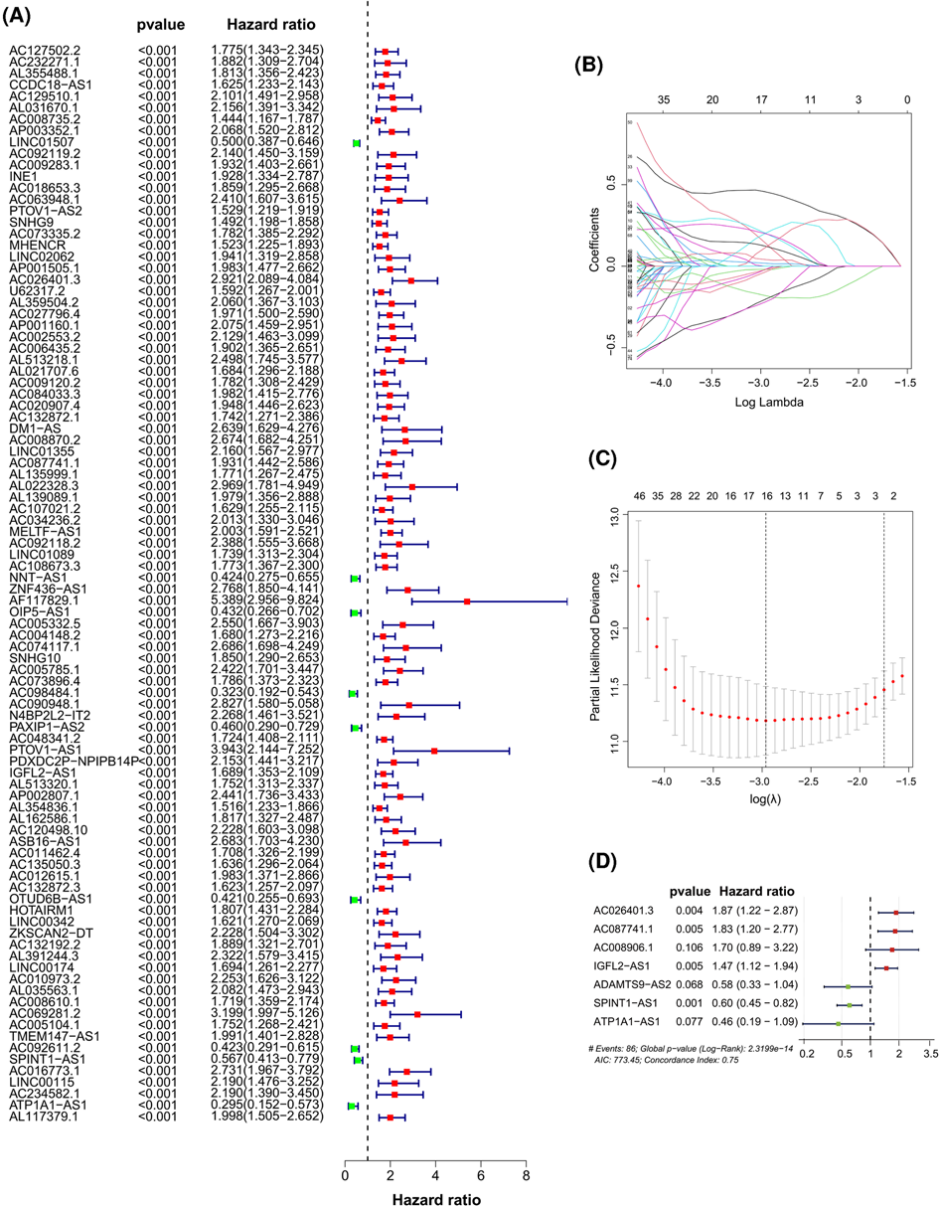
Identification of glycolysis-related lncRNAs with significant prognostic value in ccRCC (**A**) Forest plot showing the HR (95% confidence interval) and *P*-value of selected lncRNAs in the training set by univariate Cox proportional hazards analysis. (**B**) Coefficient profile plot generated for the log(λ) sequence in the training set. (**C**) Selection of optimal value of (λ) in the LASSO model by ten-fold cross-validation. (**D**) Forest plot of seven candidate glycolysis-related lncRNAs associated with ccRCC survival selected by LASSO regression analysis and construction of a prognostic model.

Patients with ccRCC were randomly assigned to the training (*n*=255) and validation (*n*=252) cohorts. A total of 297 glycolysis-related lncRNAs were identified by analyzing their coexpression with 457 glycolysis-related genes (mRNAs) using a correlation coefficient of 0.6 as the filter condition (Additional Files 1 and 2). Of these lncRNAs, 94 were related to the survival of ccRCC patients from TCGA (*P*<0.001) in the Cox proportional hazards analysis, including 85 with high risk (hazard ratio [HR] > 1) and 9 with low risk (HR < 1) (Figure 1A). We performed LASSO regression with these lncRNAs to avoid overfitting of the predicted model and estimated the prediction accuracy by ten-fold cross-validation (Figure 1B,C). Seven glycolysis-related lncRNAs with prognostic significance were further screened from the abovementioned glycolysis-related lncRNAs through multivariate Cox analysis; these were AC026401.3, AC087741.1, AC008906.1, insulin growth factor-like family member 2 antisense 1 (IGFL2-AS1), a disintegrin-like and metalloproteinase with thrombospondin type 1 motif 9 antisense 1 (ADAMTS9-AS2), serine peptidase inhibitor, Kunitz type 1 antisense 1 (SPINT1-AS1), and ATPase Na^+^/K^+^ transporting subunit α 1 (ATP1A1-AS1) (Figure 1D and Table 2).

**Table 2:** The risk model of seven glycolysis-related lncRNAs with prognostic predicting value in ccRCC

LncRNA	Coefficient	HR	HR.95L	HR.95H	*P*-value
AC026401.3	0.624714009	1.867711732	1.217147606	2.866001705	0.004244098
AC087741.1	0.601962069	1.825697432	1.202244375	2.772457234	0.004741564
AC008906.1	0.528844812	1.696970856	0.893235002	3.223910929	0.106280357
IGFL2-AS1	0.388548725	1.474838843	1.122099822	1.938463558	0.005336474
ADAMTS9-AS2	−0.540253698	0.582600429	0.326142988	1.040719169	0.067981414
SPINT1-AS1	−0.505915758	0.602953163	0.445326905	0.816372228	0.001067153
ATP1A1-AS1	−0.776596818	0.459968708	0.194289805	1.088946547	0.077366728

### Glycolysis-related lncRNA signature is a prognostic tool for ccRCC

**Figure 2:**
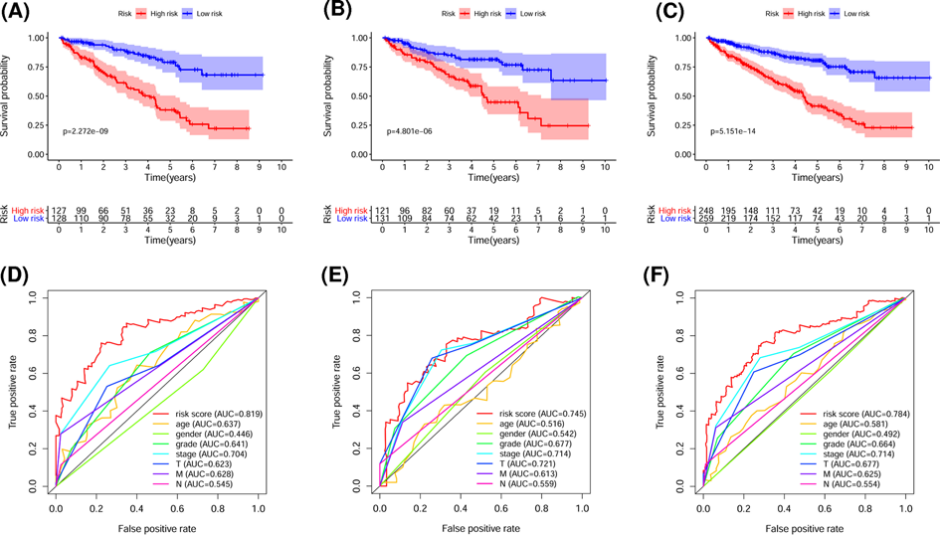
Evaluation of the predictive ability of the glycolysis-related lncRNA-based prognostic signature for ccRCC (**A–C**) Kaplan–Meier survival analysis of high- and low-risk groups in the training (A), validation (B), and total (C) sets according to the risk model and median risk score. (**D–F**) Five-year AUC for the risk model score and clinical characteristics in the training (D), validation (E), and total sets (F) based on the ROC curves.

According to the calculated median risk score, ccRCC patients in the training set were divided into high- and low-risk groups. The high-risk group showed worse OS than the low-risk group (*P*=2.272e-09) in the Kaplan–Meier survival analysis (Figure 2A). To evaluate the accuracy of the prognostic model, the same cut-off value was applied to the validation set (Figure 2B) and total set (Figure 2C). Both showed results similar to the training set (*P*=4.801e-06 and 5.151e-14, respectively), indicating that the risk score had prognostic value. The area under the ROC curve (AUC) was calculated in order to assess the specificity and sensitivity of the risk score in predicting the prognosis of ccRCC patients. The AUC of the risk score for the prognostic signature in the training set was 0.819, which was higher than that of clinicopathologic factors (Figure 2D). The AUC of the risk score for the prognostic signature was also significant in the validation set (0.745; Figure 2E) and total cohort (0.784; Figure 2F).

### Correlation between the expression of seven glycolysis-related lncRNAs and clinicopathologic characteristics

**Figure 3:**
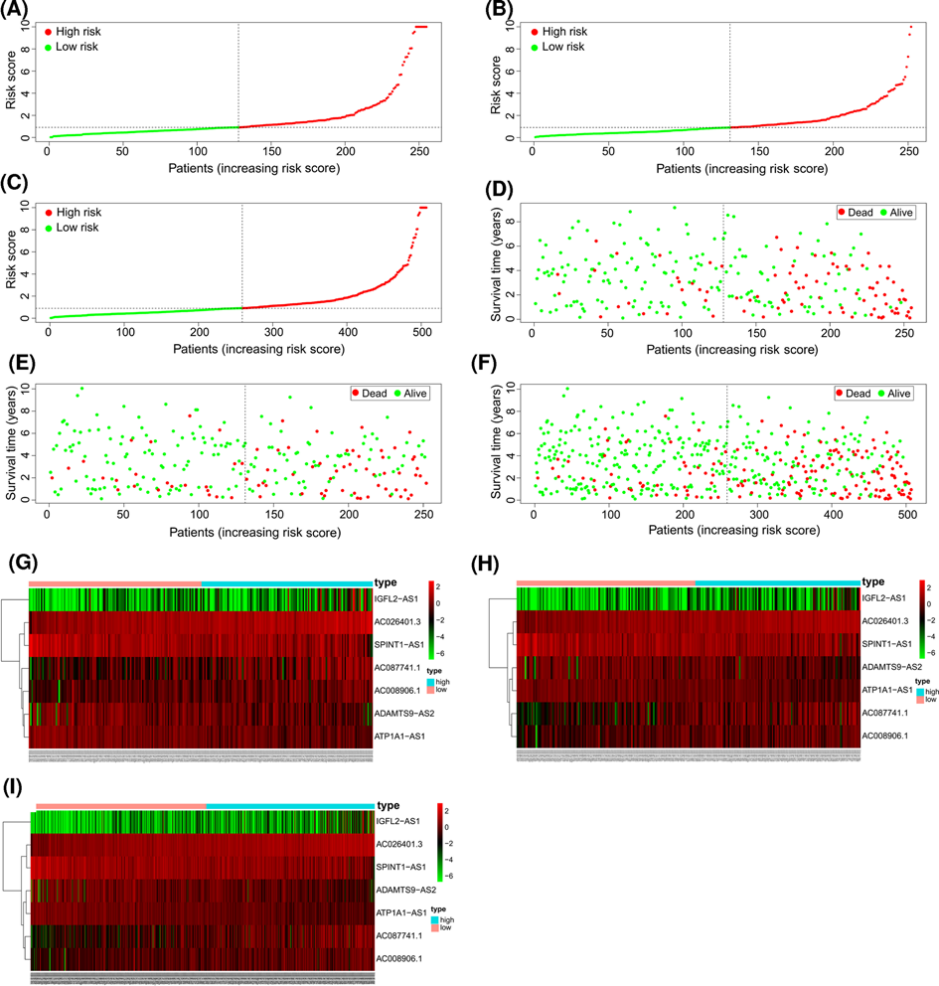
Evaluation of the prognostic risk model with the seven identified glycolysis-related lncRNAs in ccRCC (**A**–**C**) Risk curve based on the risk score of each sample in the training (A), validation (B), and total (C) sets. (**D**–**F**) Scatterplot based on the survival status of each sample in the training (D), validation (E), and total (F) sets; green and red dots represent survival and death, respectively. (**G–I**) Heatmap showing the expression levels of glycolysis-related lncRNAs in the high- and low-risk groups in the training (G), validation (H), and total (I) sets.

To further clarify the relationship between risk score and survival status of ccRCC patients, a risk curve and survival scatterplot were generated for the training, validation, and total sets (Figure 3A–F). The results showed that mortality increased with risk score. The heatmap of the expression levels of the seven identified glycolysis-related lncRNAs in ccRCC showed that AC026401.3, AC087741.1, AC008906.1, and IGFL2-AS1 were highly expressed in the high-risk group whereas the ADAMTS9-AS2, SPINT1-AS1, and ATP1A1-AS1 were up-regulated in the low-risk group (Figure 3G–I).

**Figure 4:**
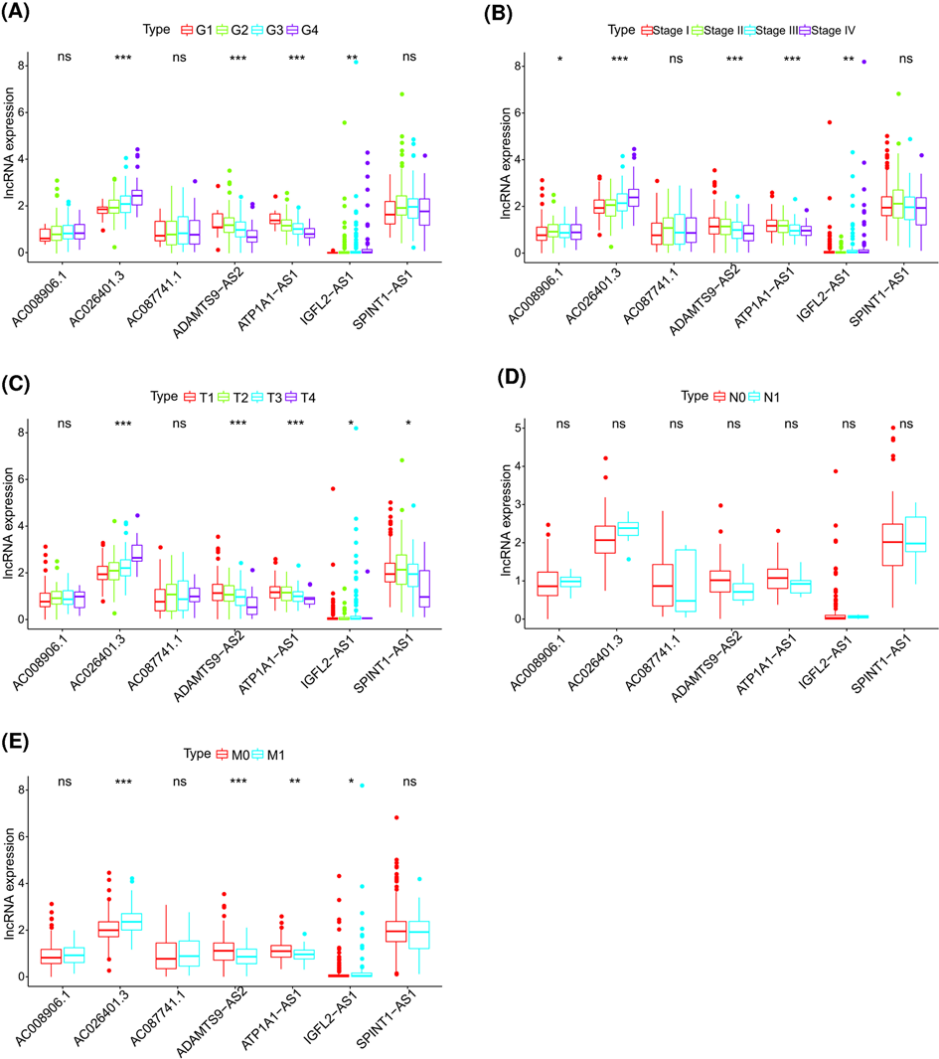
Correlation between expression of the seven identified glycolysis-related lncRNAs and clinicopathologic factors in the whole cohort (**A**) Histologic grade. (**B**) Pathologic stage. (**C**) T classification. (**D**) N classification. (**E**) M classification. *P<0.05, **P<0.01, ***P<0.001; ns, no statistical significance.

To determine whether the seven glycolysis-related lncRNAs were involved in the occurrence and development of ccRCC, we examined the relationship between their expression and clinicopathologic factors and found significant correlations with tumor grade, stage, and size as well as distant metastasis, except for lymph node metastasis. The expression of the glycolysis-lncRNAs—except for AC008906.1, AC087741.1, and SPINT1-AS1—was closely related to histologic grade (Figure 4A). Except for AC087741.1 and SPINT1-AS1, the other five glycolysis-related lncRNAs were significantly associated with pathologic stage (Figure 4B). Significant correlations were observed between most of the seven glycolysis-related lncRNAs (except for AC008906.1 and AC087741.1) and tumor size (Figure 4C). None of the seven glycolysis-related lncRNAs showed a significant correlation with lymph node status (Figure 4D). Apart from AC008906.1, AC087741.1, and SPINT1-AS1, the remaining four glycolysis-related lncRNAs were closely associated with distant metastasis (Figure 4E).

### Glycolysis-related lncRNA model predicts the prognosis of ccRCC patients

**Figure 5:**
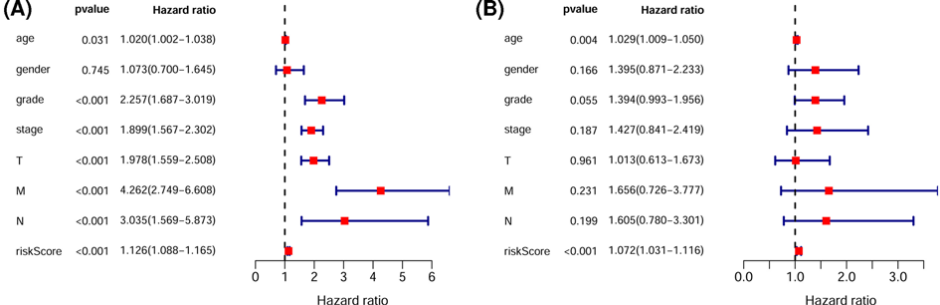
Cox regression analysis evaluating the independent prognostic value of the risk score (**A**,**B**) Univariate (A) and multivariate (B) Cox proportional hazard regression analyses of the prognostic value of the risk model score and clinical characteristics in the total set.

To further assess whether the risk model of the seven glycolysis-related lncRNAs could independently predict the prognosis of ccRCC patients, we carried out univariate and multivariate Cox regression analyses in the whole cohort. The HR (95% confidence interval) of the risk score was 1.126 (1.088–1.165) (*P*<0.001) in the univariate Cox regression analysis (Figure 5A) and 1.072 (1.031–1.116) (*P*<0.001) in the multivariate analysis (Figure 5B). Thus, the risk model of the seven glycolysis-related lncRNAs could predict the prognosis of ccRCC patients independent of clinicopathologic characteristics such as age, sex, grade, stage, tumor size, lymph node metastasis, and distant metastasis.

### Glycolysis status differs between low- and high-risk ccRCC

**Figure 6:**
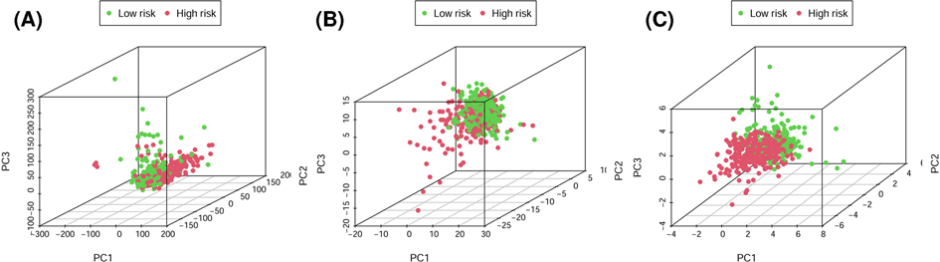
High- and low-risk groups showing different glycolytic status (**A–C**) PCA between high- and low-risk groups based on the whole genome, glycolysis-related genes, and the risk model of expression profiles of the seven identified glycolysis-related lncRNAs.

**Figure 7:**
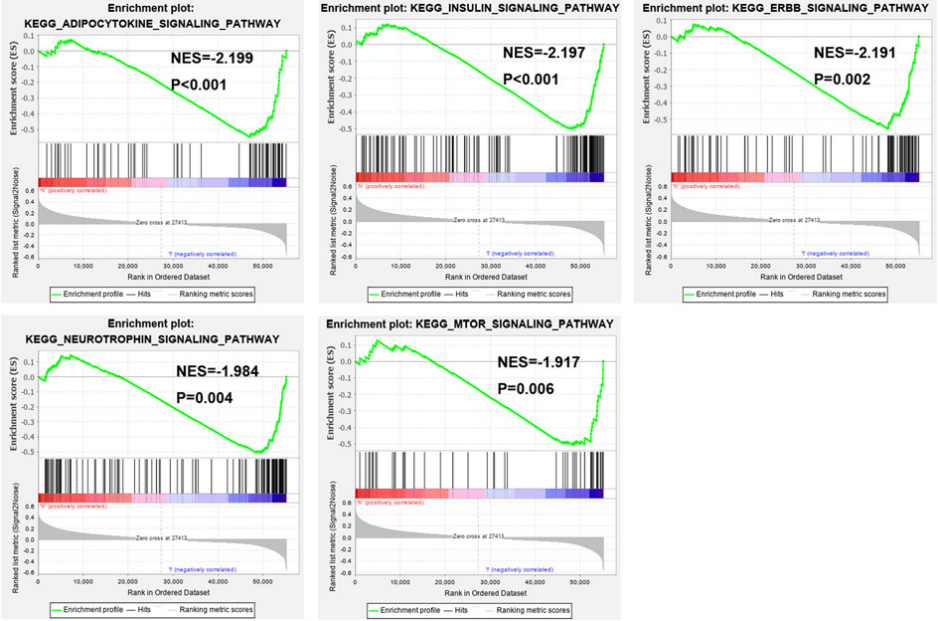
Functional enrichment analysis of the risk model of the seven identified glycolysis-related lncRNAs by GSEA

Based on the risk model established with the 7 identified glycolysis-related lncRNAs, 457 glycolysis-related genes, and whole-genome expression profiles, PCA was carried out to compare the difference between low- and high-risk groups (Figure 6). The results showed that the two groups were separated to a greater extent by the risk model than by the gene and whole-genome expression profiles; that is, ccRCC patients could be divided into high- and low-risk groups that differed significantly with respect to glycolysis status based on the risk model. In the GSEA, there were more glycolysis-related processes and pathways in the low-risk group than in the high-risk group; five glycolysis-related gene ontology terms were adipocytokine, insulin, ERBB, neurotrophin, and mammalian target of rapamycin (mTOR) signaling pathways (Figure 7). Thus, ccRCC patients with low and high risk could be distinguished based on glycolysis status using the 7-lncRNA signature.

## Discussion

As the most common subtype of renal cancer, the prognosis of ccRCC is poor as the disease has usually reached an advanced stage with distant metastasis at the time of diagnosis [26,27]. Antiangiogenic drugs, mTOR inhibitors, and immunotherapies have extended the survival of ccRCC patients. However, in most cases drug resistance develops and there is disease progression within 2 years [28]. Distant metastasis and drug resistance in ccRCC are associated with the metabolic reprogramming of cancer cells, which includes a switch to glycolysis [29–31]. As such, therapeutic strategies targeting energy metabolism may be effective in inhibiting cancer cell growth; moreover, glycolysis-related markers may have prognostic value. LncRNAs may affect the development and progression of tumors by reprogramming glucose metabolism and glycolysis in various cancers such as hepatocellular carcinoma, colorectal cancer, and gastric cancer [32–34]. Similarly, in RCC, the lncRNA FoxO-induced lncRNA 1 (FILNC1) induced by FoxO under metabolic stress was shown to inhibit renal tumor development by down-regulating c-Myc and altering glucose metabolism [35].

In the present study, we established a risk model consisting of seven glycolysis-related lncRNAs and evaluated its prognostic value for ccRCC. Some of these lncRNAs have been previously implicated in cancer including ccRCC. IGFL2-AS1 promoted gastric cancer development via a signaling axis involving the microRNA, miR-802, and cAMP-regulated phosphoprotein 19 (ARPP19) [36], and was found to predict poor prognosis in ccRCC [37]. ADAMTS9-AS2 acts as a tumor suppressor to inhibit the progression of ccRCC as well as gastric, esophageal, and bladder cancer [38–41]; and SPINT1-AS1 and ATP1A1-AS1 were predicted favorable prognosis in ccRCC [42,43]. These findings are supported by the results of our study. IGFL2-AS1 was identified as a high-risk glycolysis-related lncRNA that was associated with worse prognosis in ccRCC patients. On the other hand, ADAMTS9-AS2, SPINT1-AS1, and ATP1A1-AS1 were low-risk glycolysis-related lncRNAs that were linked to better outcome. The specific roles of the other three lncRNAs (AC026401.3, AC087741.1, and AC008906.1) in tumors are unknown and warrant further exploration. Our glycolysis-related lncRNA signature showed a significant correlation with clinicopathologic characteristics and most of the seven lncRNAs were significantly associated with histologic grade, pathologic stage, tumor size, and distant metastasis but not lymph node status. These findings suggest that the seven glycolysis-related lncRNAs contribute to the occurrence and development of ccRCC and collectively have prognostic value. Additionally, the risk score of the seven glycolysis-related lncRNA signature was more significant than that of the other clinicopathologic factors in the 5-year ROC curve analysis with the training, validation, and total sets; the 5-year AUC values for the time-dependent ROC curve were 0.819, 0.745, and 0.784, respectively, indicating that the risk model was reliable and had a better prognostic performance than clinicopathologic variables. By PCA and GSEA we determined using the glycolysis-related lncRNA risk model that the high- and low-risk groups differed in terms of glycolysis status. Thus, the difference in OS between high- vs low-risk ccRCC patients may be due to differences in energy metabolism and tumorigenic conditions induced by the seven glycolysis-related lncRNAs. The results of the GSEA showed that the seven glycolysis-related lncRNAs play an important role in ccRCC through specific signaling pathways, specifically those that are regulated by anaerobic glycolysis [44–48]. Inhibitors of mTOR signaling were shown to be effective in the treatment of ccRCC, especially in patients at an advanced stage of disease or with distant metastasis [49,50]. Our prognostic signature provides a basis for monitoring the efficacy of mTOR inhibitors or investigating their mechanisms of action in ccRCC.

Precision medicine is an important topic in tumor diagnosis and treatment. Genomic analysis can improve outcome prediction in cancer by revealing biomarkers specific to each patient [51,52]. Anaerobic glycolysis is involved in chemo- and radiotherapy resistance and tumor recurrence, which are major contributors to poor prognosis and shorter survival [53]. Given the key role of lncRNAs in regulating energy metabolism in cancer, their function in anaerobic glycolysis in cancer cells has been the focus of many studies [32,54]. A prognostic risk model based on glycolysis-related lncRNAs has been established in endometrial cancer [55]. However, the effect of glycolysis-related lncRNAs on the prognosis of ccRCC remains unclear. We developed a risk model based on 7 glycolysis-related lncRNAs that has prognostic value for ccRCC as determined with Cox and Lasso regression analyses. The signature was validated in independent datasets, demonstrating its robustness and reliability. Additionally, the signature provides potential therapeutic targets for the treatment of ccRCC patients.

This study had some limitations. Firstly, the data were got only from the TCGA datasets, and the sample was not very large. Additionally, in-depth molecular-level analyses are required to further validate our glycolysis-related lncRNA signature. We are currently examining the mechanisms by which the 7 lncRNAs affect prognosis using clinical data.

## Conclusion

In conclusion, we constructed a 7 glycolysis-related lncRNA signature for predicting the prognosis of ccRCC patients that also provides potential therapeutic targets for the individualized treatment of ccRCC patients.

## Supplementary Material

Supplementary Data

## Data Availability

All data utilized in the present study are included in this article, and all data supporting the findings of the present study are available on reasonable request from the corresponding author.

## Abbreviations

ADAMTS9-AS2: a disintegrin-like and metalloproteinase with thrombospondin type 1 motif 9 antisense 1
ATP1A1-AS1: ATPase Na^+^/K^+^ transporting subunit α 1
AUC: area under the receiver operating characteristic curve
ccRCC: clear cell renal cell carcinoma
FoxO: forkhead box O
GSEA: Gene Set Enrichment Analysis
HR: hazard ratio
IGFL2-AS1: insulin growth factor-like family member 2 antisense 1
LASSO: least absolute shrinkage and selection operator
lncRNA: long noncoding RNA
mTOR: mammalian target of rapamycin
OS: overall survival
PCA: principal component analysis
RCC: renal cell carcinoma
ROC: receiver operating characteristic
SPINT1-AS1: serine peptidase inhibitor, Kunitz type 1 antisense 1
TCGA: The Cancer Genome Atlas
